# Contribution of ^137^Cs-enriched particles to radiocesium concentrations in seafloor sediment: Reconnaissance experiment

**DOI:** 10.1371/journal.pone.0204289

**Published:** 2018-09-20

**Authors:** Takahito Ikenoue, Nobuyoshi Ishii, Masashi Kusakabe, Hyoe Takata

**Affiliations:** 1 Central Laboratory, Marine Ecology Research Institute, Iwawada, Onjuku-machi, Isumi-gun, Chiba, Japan; 2 Biospheric Assessment for Waste Disposal Team & Fukushima Project Headquarters, National Institute of Radiological Sciences, National Institutes for Quantum and Radiological Science and Technology, Anagawa, Inage, Chiba, Japan; University of South Carolina, UNITED STATES

## Abstract

Autoradiography was used to detect ^137^Cs-enriched particles in sediment samples. The contributions of ^137^Cs-enriched particles to ^137^Cs concentrations in sediment samples ranged from 9% to 64%. These experiments revealed that the variability of ^137^Cs concentrations was due mainly to the heterogeneous distribution of ^137^Cs-enriched particles in the samples. Therefore, the heterogeneous distribution of ^137^Cs-enriched particles is probably one of the main factors responsible for the temporal and spatial variations of ^137^Cs concentrations in sediment samples.

## Introduction

The Fukushima Dai-ichi Nuclear Power Plant (FDNPP) accident on 11 March 2011 resulted in the release of large amounts of anthropogenic radionuclides into the ocean and atmosphere and onto the land [1, 2]. The radioactivities of ^131^I, ^134^Cs, and ^137^Cs were particularly large among the released radionuclides [3]. Because the physical half-life of ^137^Cs is relatively long (about 30 years), it has been necessary to continue monitoring ^137^Cs contamination in the marine environment.

Radioactivity has been monitored in seafloor sediments off Fukushima and nearby prefectures regularly as a part of projects commissioned by the Japanese Ministry of Education, Culture, Sports, Science and Technology (May 2011 to March 2013) and the Secretariat of the Nuclear Regulation Authority (April 2013 to present). The ^137^Cs concentration in surface sediments generally increased with time after the FDNPP accident until the fall of 2011 [4] and then began decreasing at variable rates [5]. Use of a towed gamma ray spectrometer has revealed local ^137^Cs anomalies an order of magnitude higher than the activities on the surrounding seafloor over distances of several meters to several hundred meters [6]. Kusakabe et al. [4] have also shown large variability of ^137^Cs concentrations (170–580 Bq/kg-dry) in six surface sediment samples collected successively during one day at a sampling point in the waters off the FDNPP. Although ^137^Cs concentrations in sediments have been trending downward with time, high temporal variability of the ^137^Cs concentrations has been observed at many stations [5].

Radiocesium-bearing microparticles (CsMPs) have been found since the FDNPP accident in a variety of samples, including aerosols, soil, river water, forest leaves, vegetables, and oceanic zooplankton [7–16]. In addition to mineralogical and chemical analyses, techniques employed in these studies have included use of an imaging plate, which is well suited for identification of heterogeneously located radioactive particles. Ikenoue et al. [7] have suggested that the presence of highly radioactive particles probably explains the consistently higher ^137^Cs/^133^Cs ratios in oceanic zooplankton than in seawater. It is thus possible that the variability of radiocesium concentrations in seafloor sediments is due to the presence of heterogeneously distributed radioactive particles.

In this study, we used an imaging plate for selected samples to evaluate the impact of radioactive particles on radiocesium concentrations in seafloor sediments. Because the radioactive particles in sediments would not necessarily be identical to CsMPs in terms of chemistry and morphology, we call the particles in sediment identified by the imaging plate “^137^Cs-enriched particles (CsEPs)”. Here we report the heterogeneity of ^137^Cs concentrations in sediments due to the CsEPs.

## Material and methods

**Fig 1:**
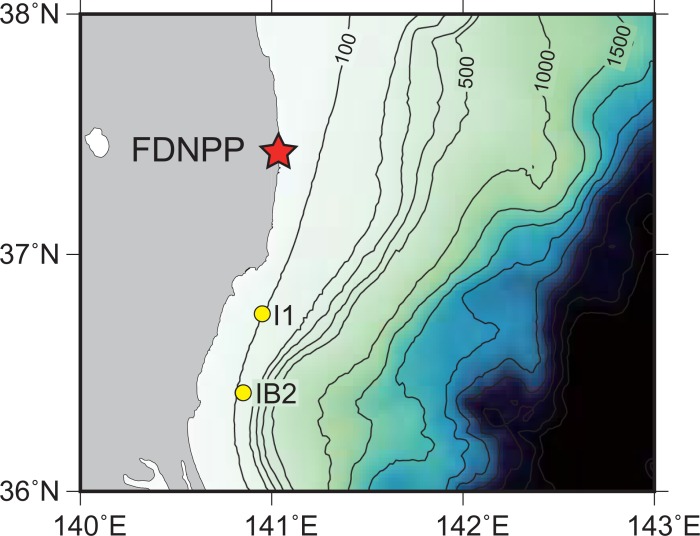
Map of the sampling locations of surface sediment. Solid yellow circles indicate the sampling stations. The red star indicates the Fukushima Daiichi Nuclear Power Plant (FDNPP).

**Fig 2:**
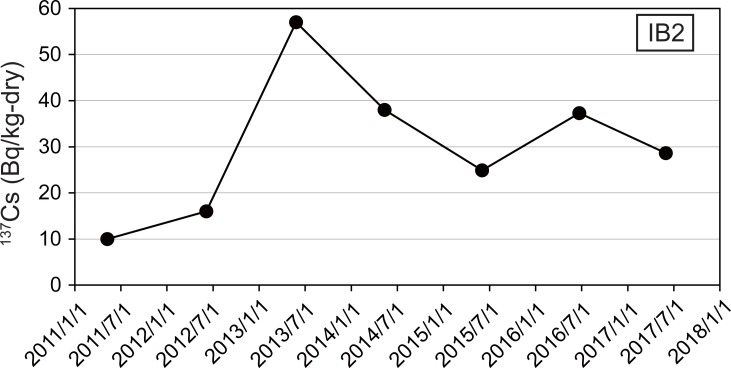
Temporal changes of the ^137^Cs concentrations in the surface sediments at Stn. IB2. Uncertainties of the data based on 1σ counting statistics are less than or equal to the size of the filled circles. The data for ^134^Cs and ^137^Cs concentrations in surface sediments used in this figure were obtained from May 2011 to June 2017 as a part of the monitoring project of the Marine Ecology Research Institute under contract with the Japanese Ministry of Education, Culture, Sports, Science and Technology (May 2011−March 2013) and the Secretariat of the Nuclear Regulation Authority (April 2013−present) [17].

Samples used for this experiment were obtained at Stn. I1 and Stn. IB2 (Fig 1, Table 1). The Cs-137 concentrations of surface (0.0–3.0 cm) sediment at Stn. I1 have been monitored since May 2011. The highest concentration at Stn. I1 was 282 Bq/kg-dry, and the rate of decline at Stn. I1 was the lowest among all stations in the waters off Fukushima and nearby prefectures as of February 2016 [5]. Furthermore, the whole-core inventory of ^137^Cs has decreased to a lesser extent with time at Stn. I1 than at other stations [5]. The surface sediment at Stn. I1 is comprised mainly of silt and clay minerals (84.4%); 6.6% of the sediment is organic matter (loss on ignition) [5]. Cs-137 concentrations of surface (0.0–3.0 cm) sediment at Stn. IB2 have also been monitored since August 1984 [17]. They did not necessarily decrease with time, showing abrupt increase by factors of 3.6 and 1.5 from June 2012 to May 2013 and from June 2015 to June 2016, respectively (Fig 2, S1 Table). The surface sediment at IB2 is comprised mainly of medium sand and fine sand (65.6%); 3.2% of the sediment is organic matter (loss on ignition) [5].

**Table 1:** Radiocesium concentrations of surface sediment samples collected at I1 and IB2.

Stn.	Location	Depth	Sampling	Sampling	Subsample	Dry weight	^134^Cs			^137^Cs			^134^Cs/^137^Cs
		(m)	date	layer (cm)	No.	(g)	(Bq/kg-dry)			(Bq/kg-dry)			
I1	36.75°N 140.95°E	96	2013/5/20	0.0–1.5	1	77	103	±	3	204	±	2	0.50
					2	78	94	±	2	190	±	2	0.49
					3	79	114	±	2	231	±	2	0.49
					Average		104	±	10	208	±	21	0.50
IB2	36.42°N 140.85°E	120	2017/5/30	0.0–1.5	-	117	9.4	±	0.4	71.9	±	1.0	0.13
				1.5–3.0	-	122	4.3	±	0.3	33.0	±	0.6	0.13
				0.0–3.0	-	170	3.8	±	0.3	28.6	±	0.5	0.13
				0.0–3.0	Calculated*		6.8	±	0.2	52.1	±	0.6	0.13

*Radiocesium concentrations were calculated based on the individually measured radioactivity of subsamples (0.0–1.5-cm and 1.5–3.0-cm layers).

The surface sediment samples were retrieved with a multiple corer equipped with eight plastic tubes (No. 5173-A, Rigo Co., Ltd., Tokyo, Japan) at Stn. I1 and with a box corer (No. 5153, Rigo Co., Ltd., Tokyo, Japan) at Stn. IB2. Since the samples of each sampling station were collected in separate surveys, sampling methods were different; however, only the bottom sediments whose surface were not disturbed were collected, and so there was no difference in the quality of the samples due to the difference in sampling methods. In the case of the samples from Stn. I1, surface sediments (0.0–1.5-cm depth) from the eight cores were combined together onboard the research vessel. In the case of the samples from Stn. IB2, several plastic tubes were inserted into the box corer to get sub-core samples. The sub-core samples were cut into three different depth intervals: 0.0–1.5 cm, 1.5–3.0 cm, and 0.0–3.0 cm to observe the vertical distribution of radioactive cesium. The samples from each depth interval were combined together onboard the research vessel. In the laboratory on land, each sample was dried at 105°C, ground in a mortar, sieved through a screen (mesh size <2 mm), and then pulverized to a homogeneous powder in a table-top grinder.

The dried bulk sample from Stn. I1 was divided into three subsamples to observe the variations in radiocesium activity among the subsamples. Each subsample was placed in a U8 container to measure its radiocesium activity. The dried bulk samples from Stn. IB2 were also placed in U8 containers without subsampling. The radioactivities of ^134^Cs and ^137^Cs in each sample were measured with a coaxial type Ge detector over counting periods of tens of thousands of seconds. The radioactivity in the samples was decay-corrected to the sampling date.

Autoradiography was applied to the two samples with the lowest and highest ^137^Cs concentration among the three subsamples from Stn. I1 within the 0.0–1.5-cm layer, i.e., No. 2 and No. 3, and to the sample from Stn. IB2 within the 0.0–1.5-cm layer. The samples were spread in plastic bags. A 20 x 40 cm imaging plate (BAS-MS 2040, Fujifilm) in contact with the bagged sample was exposed for three days in a cassette (BAS cassette2 2040, Fujifilm). The exposed imaging plate was scanned on a Fluor Image Analyzer (FLA-5100, Fujifilm) using a 635 nm laser. Scanned images (img format) were converted to tiff format using a software (Multi Gauge Ver. 2.3, Fujifilm). The white and black contrast of the autoradiographic image was adjusted so that CsEPs, which appeared as black dots on an image, could be discriminated from the remaining CsEP-free fraction of a sample.

**Fig 3:**
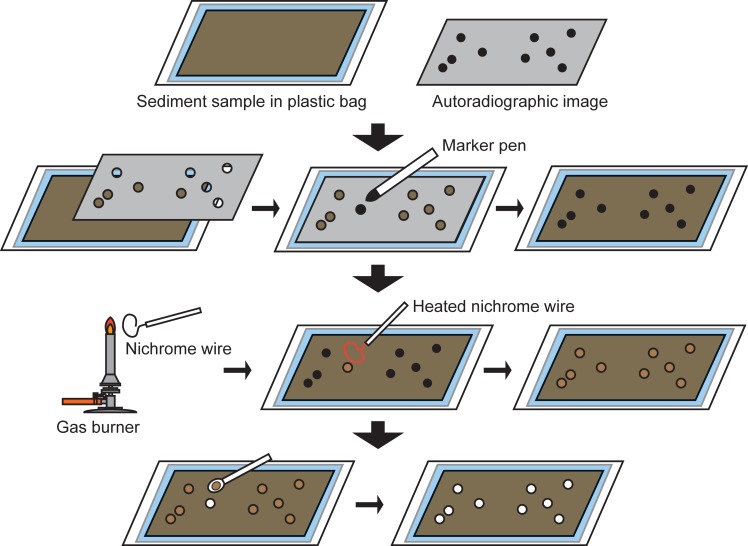
Schematic diagram of procedure to remove CsEPs from the sediment sample in plastic bag.

The autoradiographic image was used for the removal of CsEPs from the sample as follows (Fig 3). Holes were punched by the point of a pencil at the black spots on the full-sized, printed autoradiographic image. The image was then superimposed on the sediment sample in the plastic bag, and the hole was painted in black with a marker pen. The black points on the bag were removed with a nichrome wire heated with a gas burner, and the CsEP was removed with a small spoon inserted through the holes of the plastic bag. The CsEP was removed with small amount of the surrounding sediment so that none of the CsEP was left behind. In this study, the sediment sample with its CsEP removed was designated as the remaining sample. The remaining sample was further divided into five subsamples, and the dry weight of each subsample was measured. Each subsample was then inserted into a U8 container, and the radioactivities of ^134^Cs and ^137^Cs were measured with a coaxial type Ge detector over a counting period of tens of thousands of seconds to confirm that there was no significant variation of radioactive Cs concentrations in the remaining samples. The radioactive Cs concentrations in the samples were decay-corrected to the sampling date.

## Results and discussion

Table 1 shows the radiocesium concentrations and ^134^Cs/^137^Cs activity ratios of three subsamples separated from the surface sediment sample of Stn. I1. Although the concentrations of ^134^Cs and ^137^Cs in subsamples ranged from 94 to 114 Bq/kg-dry and from 190 to 231 Bq/kg-dry, respectively, their ratio was relatively constant at 0.49–0.50, very similar to the theoretical ratio of 0.50 (on 20 May 2013) due to the different half-lives of ^134^Cs and ^137^Cs and their activity ratio of 1.0 at the time of the FDNPP accident [18]. The maximum value of ^137^Cs concentration was higher than the minimum by 22%, which was a significant difference even though small sample number and error derived from counting statistics were taken into account. The average fraction of the radiocesium in the sediment organic component in the offshore-Fukushima region was reported to be 5.7 ± 3.0% of the bulk sediment inventory [19]. Most of the radioactive Cs was thus in the sediment mineral fraction. It is well known that Cs has strong affinity with fine minerals [20, 21]. Therefore, one of the main factors to control the variability of the radiocesium concentrations in sediment mineral fraction is grain size distribution. However, it was not the case for the variability of the radiocesium concentrations in this study as the three subsamples came from a homogenized sediment sample.

**Fig 4:**
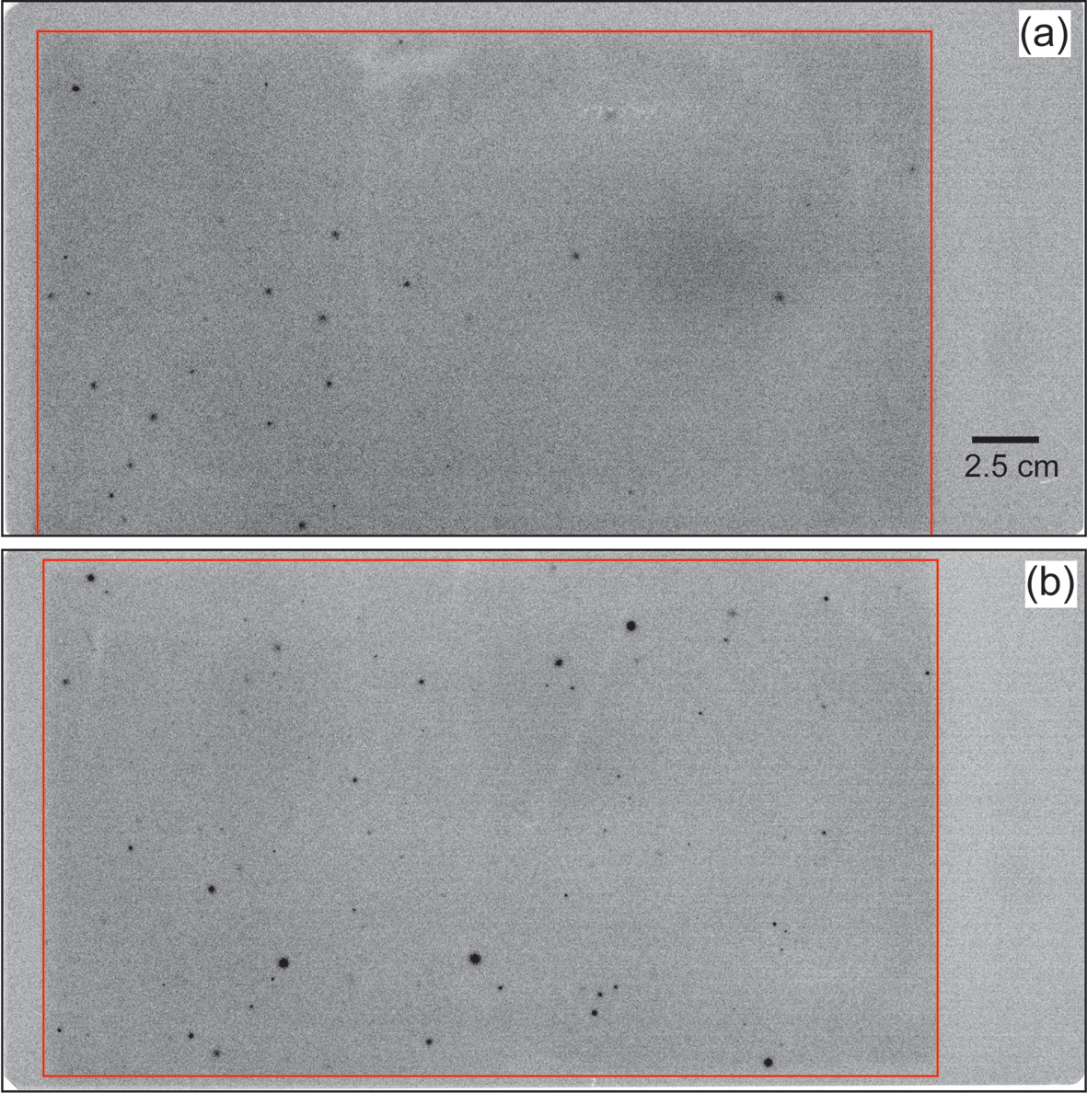
**Autoradiographic image of subsamples No 2 from Stn. I1 (a) and No. 3 from Stn. I1 (b).** The area surrounded by the red line corresponds to the sediment samples.

**Fig 5:**
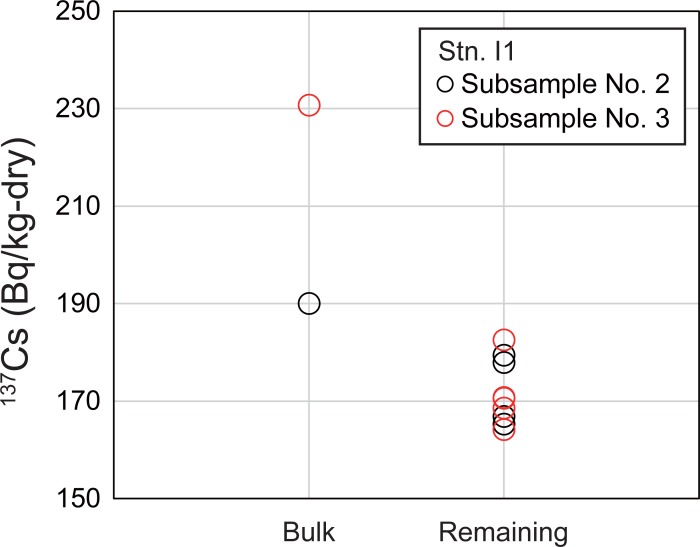
Comparison of dispersion of ^137^Cs concentrations in bulk samples and remaining samples from Stn. I1. ^137^Cs concentrations in subsample No. 2 are shown as red circles, and those in subsample No. 3 are shown as black circles.

Autoradiography was applied to subsamples No. 2 and 3 from Stn. I1 sediment. The CsEPs in the samples appear as black spots in Fig 4. In subsamples No. 2 and No. 3, 28 and 71 black spots, respectively, were recognized. Table 2 shows the radiocesium concentrations of the remaining samples. The radiocesium concentrations of each remaining sample were determined from the average and standard deviation of the five subsamples (Table 2). The ^137^Cs concentration of the remaining sample was lower than that of the bulk sample by 9% for subsample No. 2 and by 26% for subsample No. 3 (Fig 5). The variability of radiocesium concentrations in the bulk sample could thus be accounted for by the heterogeneous distribution of CsEPs in the samples.

**Table 2:** Radiocesium concentrations of remaining samples.

Stn.	Subsample	Remaining	Dry weight	^134^Cs			^137^Cs			^134^Cs/^137^Cs
		sample No.	(g)	(Bq/kg-dry)			(Bq/kg-dry)			
I1	No. 2	1	15.79	87	±	5	171	±	3	0.51
		2	15.30	82	±	4	165	±	3	0.50
		3	12.92	81	±	5	167	±	3	0.48
		4	12.64	92	±	5	179	±	3	0.51
		5	17.18	94	±	4	178	±	2	0.53
		Average		87	±	6	172	±	6	0.51
	No. 3	1	14.51	87	±	5	183	±	3	0.47
		2	11.97	88	±	5	171	±	3	0.52
		3	14.29	88	±	2	169	±	1	0.52
		4	14.41	82	±	5	164	±	3	0.50
		5	12.16	78	±	5	171	±	3	0.46
		Average		85	±	5	171	±	7	0.49
IB2	0.0–1.5 cm	1	21.66	3.3	±	0.6	24	±	1	0.14
		2	24.33	3.5	±	0.4	24	±	1	0.15
		3	24.11	3.2	±	0.5	27	±	1	0.12
		4	22.12	3.3	±	0.5	27	±	1	0.12
		5	22.00	4.5	±	0.6	28	±	1	0.16
		Average		3.6	±	0.5	26	±	2	0.14

**Fig 6:**
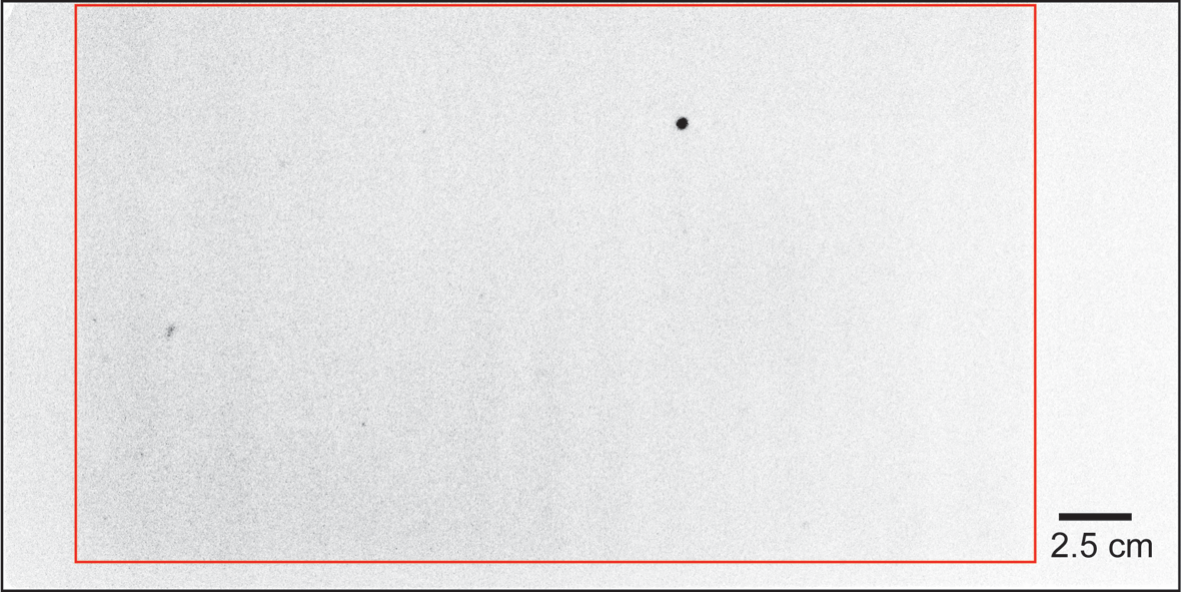
Autoradiographic image of the sediment sample from Stn. IB2. The area surrounded by the red line corresponds to the sample.

**Fig 7:**
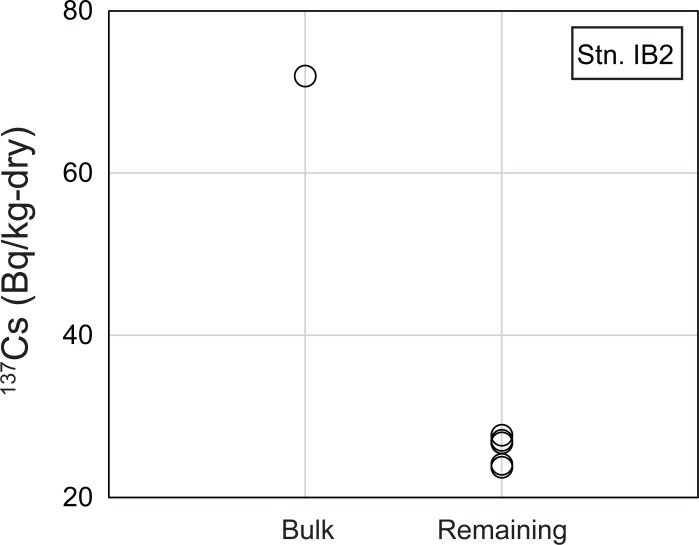
Comparison of ^137^Cs concentrations between the bulk sample and remaining sample from Stn. IB2.

At Stn. IB2, the ^137^Cs concentration measured in the 0.0–1.5-cm layer (72 Bq/kg) was 2.2 times higher than measured in the 1.5–3.0-cm layer (33 Bq/kg) (Table 1). On the other hand, the ^137^Cs concentration in the 0.0–3.0-cm layer sample was 29 Bq/kg, which was lower than either 0.0–1.5 cm or 1.5–3.0 cm layer sample (Table 1). In addition, the ^137^Cs concentration in the 0.0–3.0-cm layer calculated by summing the concentrations in 0.0–1.5-cm and 1.5–3.0-cm layer samples was 52 Bq/kg, which was 1.8 times higher than the measured ^137^Cs concentration in 0.0–3.0-cm layer sample (Table 1). This result implies that the distribution of radiocesium was heterogeneous in the samples. Their ^134^Cs/^137^Cs activity ratios were relatively constant at 0.13, comparable to 0.14, the ratio expected from the theoretical decay of the ^134^Cs/^137^Cs activity ratio since the time of the FDNPP accident. The autoradiograph of the sample at Stn. IB2 showed two black spots (Fig 6). The ^137^Cs concentration of the Stn. IB2 remaining sample was lower than that of bulk sample by 64% (Fig 7; Table 2). In other words, two-thirds of the ^137^Cs in the sediment was associated with CsEPs. The patchy distribution of high radiocesium concentrations in the bulk sample from Stn. IB2 was therefore due mainly to the heterogeneous distribution of CsEPs in the samples.

Under the assumption that the total weights of CsEPs were negligible compared to the total sample weights (shown in Table 1), the weights of the remaining and bulk samples should be the same. We used the following equation to estimate of the radioactivity of radiocesium derived from CsEPs:

$$A_{p}{=A}_{\mathrm{blk}}{-A}_{\mathrm{rem}}$$ (1)

where A_p_, A_blk_, and A_rem_ are the radioactivity of radiocesium in the CsEPs, bulk sample, and remaining, respectively.

Table 3 shows the A_blk_ and A_rem_ in the subsamples from Stn. I1 and the sample from Stn. IB2. The radioactivity of ^134+137^Cs was 3.7 times higher in the particles from subsample No. 3, which contained the most black spots, than in those from subsample No. 2. However, the radioactivity was not in direct proportion to the number of black spots because the radioactivities of individual CsEPs were variable, as shown in Fig 4. Indeed, only two black spots in the IB2 sample contained more ^134+137^Cs radioactivity than 71 black spots in subsample No. 3 from Stn. I1. If it is assumed that the number of black spots corresponds to the number of CsEPs, then the average ^137^Cs radioactivity per CsEP was 0.05 ± 0.02 Bq in subsample No. 2 from Stn. I1, 0.07 ± 0.01 Bq in subsample No. 3 from Stn. I1, and 2.7 ± 0.1 in the sample from Stn. IB2.

**Table 3:** Radioactivities of radiocesium in bulk sediments, remaining samples, and CsEPs.

Stn.	Subsample	Bulk sample*						Remaining sample**						CsEPs						
		^134^Cs			^137^Cs			^134^Cs			^137^Cs			^134^Cs			^137^Cs			^134^Cs/^137^Cs
		(Bq)			(Bq)			(Bq)			(Bq)			(Bq)			(Bq)			
I1	No. 2	7.3	±	0.2	14.8	±	0.1	6.8	±	0.5	13.4	±	0.5	0.5	±	0.5	1.4	±	0.5	0.37
	No. 3	9.0	±	0.2	18.3	±	0.1	6.7	±	0.4	13.6	±	0.5	2.3	±	0.4	4.7	±	0.6	0.49
IB2	0.0–1.5 cm	1.10	±	0.04	8.4	±	0.1	0.4	±	0.1	3.0	±	0.2	0.7	±	0.1	5.4	±	0.2	0.13

*Radioactivities were calculated based on the radiocesium concentrations and dry weights in Table 1.

**Radioactivities were calculated based on the average of the radiocesium concentration in remaining samples in Table 2 and the dry weight of bulk samples in Table 1.

The CsEPs in Stn. I1 sample contributed to the increase (9–26%) of the radioactivities of ^137^Cs in the samples because there were substantial numbers of CsEPs in the samples. However, the average activities of ^137^Cs per CsEP at Stn. I1 were one to three orders of magnitude lower than those reported in aerosol samples (0.66–3.27 Bq/particle [8]), soil particles on the land (67.1 Bq/particle [9]), and particles in river water (0.1 to 2.8 Bq/particle [10]). In contrast, the average activities of ^137^Cs per CsEP at Stn. IB2 was comparable with the radioactivity of CsMPs in the aerosol and river water samples [8], [10]. The temporal fluctuation of ^137^Cs concentration shown in Fig 2 can thus be ascribed to a few particles. For example, addition of 2.3 CsEPs to 150 g of sediment would account for the increase of 41 Bq/kg-dry from 5 June 2012 to 26 May 2013.

As mentioned above, the abundance of CsEPs should be a main candidate for factors to control the temporal and spatial variation of radiocesium concentration in sediment samples. In addition to the CsMPs in the aerosol [8] and river water [10], such particles have been found in the suspended matter of the coastal waters around the FDNPP [22]. Although their pathway to the bottom sediment has not been clear yet, they should be eventually transported to the sea floor as sinking particles. And the fate of the particles in the bottom sediment is yet to be studied in detail. The temporal fluctuation of ^137^Cs concentration in surface sediment might be owing to two mechanisms (1) sporadic lateral migration of bottom sediment containing CsEPs through a cycle of deposition and suspension, and (2) sparse distribution of CsEPs in the bottom sediment.

The results of monitoring ^137^Cs concentrations in seafloor sediments off Fukushima and nearby prefectures have sometimes revealed concentrations that deviate by several folds from the average regression trend [5]. These deviations cannot be fully explained by particles such as those found in this study. In future studies, the distribution of CsEPs and their effects on seafloor sediments need to be investigated over a wider area. In addition, because the heterogeneity of the CsEP activities in sediment samples seems to be the main determinant of the fluctuations of Cs concentrations, greater numbers of samples that are also larger in mass may be required to get a more accurate estimate of the inventory of ^137^Cs in the marine environment.

## Supporting information

S1 Table Radiocesium concentrations of surface sediment samples at Stn. IB2. (XLSX)
